# Subtle variations in mobbing calls are predator-specific in great tits (*Parus major*)

**DOI:** 10.1038/s41598-019-43087-9

**Published:** 2019-04-25

**Authors:** Nadine Kalb, Fabian Anger, Christoph Randler

**Affiliations:** Department of Biology, Eberhard Karls Universität Tübingen, 72076 Tübingen, Germany

**Keywords:** Behavioural ecology, Evolutionary ecology

## Abstract

Many species are known to use vocalizations to recruit con- and heterospecifics to mobbing events. In birds, the vocalizations of the Family Paridae (titmice, tits and chickadees) are well-studied and have been shown to recruit conspecifics and encode information about predation risk. Species use the number of elements within a call, call frequency or call type to encode information. We conducted a study with great tits (*Parus major*) in the field where we presented taxidermy mounts of two predators of different threat levels (tawny owl, *Strix aluco*, and sparrowhawk, *Accipiter nisus*) and compared the mobbing calls of these two contexts. We hypothesized, based on results of studies in other paridae species, that tits vary the number or type of elements of a call according to predatory context. We found great tits to vary the number of D elements and the interval between those elements. Great tits produced significantly longer D calls with more elements and longer intervals between elements when confronted with a sparrowhawk (high-threat) compared to a tawny owl (low-threat) mount. Furthermore, birds produced more D calls towards the high-threat predator. This suggests that the basic D calls are varied depending on threat intensity.

## Introduction

Animals transmit information in various ways with vocal, visual and olfactory signals being the most common ones. Visual signals are normally visible over short distances due to their physical properties. Vocalizations in contrast can be transmitted over longer distances and are therefore suitable to transmit information also to individuals that are not in visual contact to the sender^1^. Animal vocalizations have been studied in a wide variety of taxa and some vocal signals in avian and mammalian species are even known to encode information about environmental factors such as the presence of predators and food^2–5^.

Many bird species produce alarm or mobbing calls after a predator has been detected^6–8^. Usually, alarm calls are produced to inform others about a threat that causes them to flee or hide, mobbing calls on the other hand are intended to attract hetero- and conspecifics to join a mobbing flock^9–11^. During mobbing, songbirds produce distinct mobbing calls, move towards the predator and display stereotype behaviors to recruit others and deter the predator^12–14^. Additionally, calls can transmit information about a predator’s type^15^, size^16^ and distance^17^. Although moving towards the predator while mobbing seems controversial in terms of immediate predation risk, it ultimately can hold the benefit of chasing the predator away^18–20^. Further, exhibiting mobbing behavior and alarm calling at or in the nest can increase the fitness of incubating females and their young^21,22^.

In birds, the mobbing behavior of titmice, tits and chickadees (Family Paridae) is especially well-studied. Paridae species do not only transmit information in their calls about the presence of a predator, but also about its threat level^23–26^. Information about a predator can be encoded by an increased call intensity, a variation in syllable number, syllable duration or call type. Some species use only one of these possibilities and others a combination of some or even all ways, whereby more dangerous predators usually elicit a stronger response^23,27^. Tufted titmice (*Baeolophus bicolor*) increase the total number of D notes per time unit towards more threatening predators^24^. Black-capped chickadees (*Poecile atricapillus*) produce calls with more D notes and decrease the duration of the first D note as well as the time between the first and the second D note, when confronted with smaller and more dangerous predators^16^. Similar Carolina chickadees (*Poecile carolinensis*) produce more ‘chick” and fewer ‘dee” notes in the presence of a larger, low-threat predator, whereas smaller and higher-threat predators elicit fewer ‘chick’ and more ‘dee’ notes^28^. Japanese great tit parents (*Parus minor*) produce distinct alarm calls when confronted with three of their main nest predators^15,26,29^. They produce jar calls solely in response to Japanese rat snakes (*Elaphe climacophora*) and vary the number of ‘chicka’ calls as well as the number and type of notes within ‘chicka’ calls to further discriminate between Japanese marten (*Martes melampus*) and jungle crow (*Corvus macrorhynchos*)^29^. Adults show different predator-searching^30^ and nestlings predator-avoidance^15,31^ responses according to the respective alarm calls. Incubating great tit females give hissing calls when an intruder enters the nest box^21^ and breeding pairs produce churring calls (D calls) when a predator is close to the nest^22^. Non-breeding great tits decrease the proportion of calls containing chirp elements and increase the propensity to produce jar/rattle calls to distinguish between threatening predators and a control^23^. To discriminate between predators of different threat-level great tits increase their call rate in response to higher threats^23,32^.

There are numerous studies that investigated how passerines encode information about predators in their calls, but most studies focused on changes in calling rate and call types in response to different predators. Hence, our goal was to investigate if wild-living great tits might use fine-scale acoustic variations in their mobbing calls as an additional way of encoding information about predatory threats. We recorded mobbing calls of great tits when confronted with taxidermy mounts of tawny owl (*Strix aluco*) and sparrowhawk (*Accipiter nisus*). Dutour, *et al*.^33^ showed that mobbing behavior in passerines increases with the prevalence in a predator’s diet. Hence, we presented great tits two avian predator species that greatly differ in the proportion of great tits in their diet and consequently pose different predation risk to this species. Both predators are common in southwest Germany in general and in our study area in particular^34,35^ and are known to prey on small passerine birds including great tits^36–39^. Sparrowhawks are high-threat predators for great tits as they are diurnal and small birds, including great tits, make up the most part of their diet^39^. Tawny owls on the other hand are most active at twilight and night and mainly prey on small mammals^37,38^ and can therefore be considered as low-threat predator for great tits. Curio, *et al*.^36^ found that great tit parents feeding fledglings respond more strongly (shorter mean minimum and average distance) during mobbing towards sparrowhawks, which have a higher “predator pressure” than towards tawny owl. We hypothesized that great tits do not only use call rate and type^23^ but also some fine scale acoustic measures^16^ to discriminate between the two predators.

## Results

### Call types

We compared the number and call types (D call, chirp, tonal and jar) produced towards the two predators of different threat level (sparrowhawk versus tawny owl). Season had not effect on any of the measured variables (all p > 0.05). Great tits produced chirp, tonal, and D calls in response to both predators. Jar calls were only recorded in four locations in response to the tawny owl. Among the four call types, D calls were the most frequently given ones (tawny owl: 83.12%, sparrowhawk: 94.12%). The total number of calls, jar, chirp and tonal calls was not significantly affected by predator type or conspecifics (all p > 0.05). However, predator type (F = 5.537, df = 1,34, p = 0.025) and number of conspecifics (F = 6.811, df = 1,34, p = 0.013) had an effect on the number of produced D calls in three minutes. The number of D calls was significantly higher in the sparrowhawk treatment compared to the tawny owl treatment and increased with increasing number of conspecifics. Call rate (D calls/individual/minute) was also affected by predator type (F = 5.402, df = 1,1, p = 0.026), but not by the number of conspecifics (F = 3.176, df = 1,1, p = 0.084). The call rate was higher in response to the sparrowhawk (mean ± SE, 15.03 ± 2.75) compared to the tawny owl (7.97 ± 1.20). We also found an effect of predator type on the mean number of elements in D calls (F = 5.767, df = 1,34, p = 0.022). Here, great tits produced calls with more D elements exposed to the sparrowhawk compared to the tawny owl mount.

### D call features

**Figure 1:**
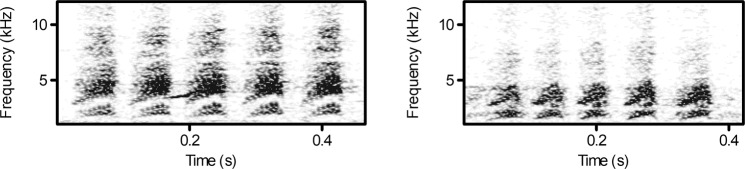
Sonogram showing a D call with 5 elements in response to a sparrowhawk mount (left) and a tawny owl mount (right). Sparrowhawk mobbing calls have a longer duration (s) and longer intervals between elements (s) than calls in response to the tawny owl.

**Figure 2:**
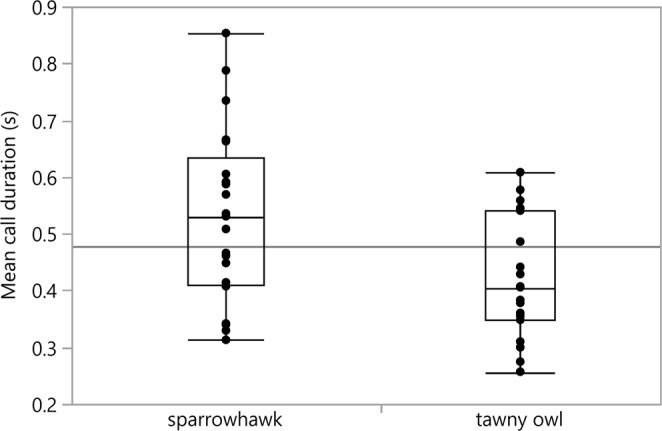
Mean call duration (s) depending on the predator type model. Call duration is significantly longer in calls towards the sparrowhawk than towards the tawny owl.

**Figure 3:**
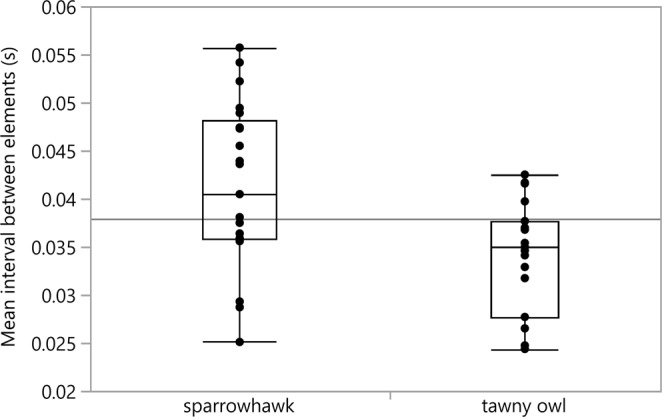
Mean interval between D elements (s). Birds produced calls with longer intervals between elements when confronted with a sparrowhawk model compared to the tawny owl model.

We found a significant effect of predator type on the mean duration of D calls (F = 6.167, df = 1,34, p = 0.018). Great tits produced longer D calls towards the sparrowhawk (0.531 ± 0.033) than towards the tawny owl mount (0.419 ± 0.024) (Figs 1, 2). Predator type as well had an effect on the number of D elements within a call (F = 4.389, df = 1,34, p = 0.044) as great tits produced calls with more elements towards the high-threat predator (7.095 ± 0.391) than towards the low-threat predator (6.063 ± 0.371). Moreover, predator type affected the mean interval between elements (F = 4.405, df = 1,34, p = 0.043), whereby the interval between elements was longer when confronted with a sparrowhawk (0.041 ± 0.002) compared to the tawny owl (0.034 ± 0.001) (Figs 1, 3). The mean duration of elements was not affected by predator type (F = 0.796, df = 1,34, p = 0.379). The number of conspecifics or season had no significant effect on any of our measured parameters (all p > 0.05).

In respect of acoustic features, there was no significant effect of predator type on the mean peak frequency (F = 0.124, df = 1,29, p = 0.728), maximum frequency above −30 dB (F = 0.282, df = 1,29, p = 0.599), number of peak above −10 dB (F = 0.604, df = 1,29, p = 0.443), interval between overtones (F = 0.075, df = 1,29, p = 0.786), bandwidth at −10 dB (F = 0.005, df = 1,13, p = 0.945) or bandwidth at −30 dB (F = 0.575, df = 1,29, p = 0.454). The number of conspecifics also had no effect on any given parameter (all p > 0.05).

## Discussion

This study shows context-dependent variation in vocalizations in a common passerine, the great tit. Great tits responded differently towards a low-threat (tawny owl) and high-threat (sparrowhawk) predator.

Irrespective of the predator type great tits produced chirp, tonal and D calls, whereby the majority of calls were D calls. We found great tits to increase call rate and the number of D calls in three minutes when exposed to a high-threat compared to low-threat predator. This is in line with findings by Carlson, *et al*.^23^ who found that British great tits vary calling rate to discriminate between high- and low-threat predators. Moreover, Templeton, *et al*.^16^ showed that black-capped chickadees produce significantly more mobbing calls, particularly more D syllables, towards small high-threat predators than towards larger predators.

The number of D calls was not only affected by predator type but also by the number of conspecifics present in a radius of 50 meter. This might be explained by the fact that we did not record single individuals but all great tits participating in a mobbing event under natural conditions. Hence, an increase in the number of D calls might be explained by two factors: (i) flock size, i.e. single individuals might call more frequently when accompanied by conspecifics compared to when being alone or (ii) an additive affect, i.e. more individuals participate in mobbing leading to an increased number of calls. Since call rate (i.e. D calls/individual/minute) was not significantly affected by the number of conspecifics, the latter explanation seems to be more likely.

The more individuals join a mobbing flock and exhibit mobbing behavior the higher is the chance of successfully driving the predator away^19^. However, joining a mobbing flock also holds the risk of getting captured or giving away the location of the nest^40–42^. Therefore, if individuals assist in mobbing or stay in safety might depend on the community composition, i.e. whether it is accompanied by familiar or related individuals or not. The alarm calling and mobbing behavior of some birds, including great tits, is known to be affected by the familiarity with con- and heterospecifics^43–49^. The effect of number of conspecifics on D call number, irrespective of predator type, could possibly also be explained by such familiarity effects. Great tits live in monogamous pairs during breeding season and join flocks during winter^50,51^. In some of our study locations we observed up to 30 breeding pairs per square-kilometer, which makes some kind of familiarity among breeding pairs likely. Great tit breeding pairs are known to be more likely to join the nest defense of familiar neighbors than of unfamiliar ones^46^ and male wintering great tits give alarm calls more frequently when being with their mates or when being accompanied by permanent flock members in their home range^48^. Hence, it seems possible that the number of birds joining a mobbing flock and consequently the number of calling individuals is also affected by familiarity among great tits living in a specific area. Further, anti-predator responses in great tits can be related to the personality of the caller^21,22^, i.e. some individuals are repeatedly willing to take higher risks during predator defense than others. Hence, one might expect that both the number of calls and the number of mobbing individuals increases in communities with a high proportion of bold individuals. However, if and how personality and familiarity affect the composition of a mobbing flock and the mobbing behavior therein remains to be tested.

We found great tits to not only vary the number of D calls but also the duration of those calls. Great tits produced longer D calls towards the high-threat compared to the low-threat predator. Birds can alter the duration of calls by varying either one or a combination of the following variables (1) the number of elements of a call, (2) the duration of elements or (3) the intervals between elements. In our study the difference in call duration resulted from a variation in element number and the interval between elements. Calls towards the high-threat predator had more elements than calls in response to the low threat predator. Such a variation in element number according to predator threat is widespread in paridae species, including great tits^16,23,24^. However, to our best knowledge, we are the first to reveal that great tits also use a variation of the intervals between elements to encode information about predator threat. Great tits produced calls with longer intervals between elements in response to the sparrowhawk than in response to the tawny owl. Templeton, *et al*.^16^ as well found such a variation in black capped-chickadees, which decrease the duration of the first D note as well as the interval between the first and second D note when confronted with smaller, more dangerous predators. Acoustic analysis of mobbing calls in American crows (*Corvus brachyrhynchos*) also suggest that calls with a longer duration, higher rate and shorter intervals between caws encode a higher predation risk^52^. These and our results combined indicate that subtle variations in the interval between elements of one call type might hold the potential to encode numerous information about predator threat without changing call type. Future work could investigate if great tits use variations in the interval between elements to discriminate between different predator types (e.g. terrestrial versus aerial). In addition, future research is necessary to investigate if great tits are able to recognize such subtle variation in call structure and adapt their behavior accordingly.

In our study, great tits produced jar calls only in four out of 40 locations and only in response to the tawny owl mount. Japanese great tits (*Parus minor*) produce jar calls as referential signal specifically in response to snakes and ‘chicka’ calls (including D calls) for avian and mammalian nest predators^15,29,31^. In *Parus major*, however, call types exclusively used only in specific predation contexts have, so far, not been found. Our finding also does not indicate such functional referential signalling as it is in contrast to findings by Carlson, *et al*.^23^ where great tits increased the propensity to produce jar/rattle calls when confronted with an avian predator compared to the control. Such differences might be due to geographical variation or differences in the experimental design. Carlson and colleagues presented both predators (sparrowhawk, common buzzard, *Buteo buteo*) at each site. We used only one predator per site and hence cannot account for possible inter-individual differences in calling behavior. Krams, *et al*.^21^ found that incubating great tits produce hissing calls when confronted with a nest intruder. Surprisingly, females differ in their propensity to give hissing calls, which might reflect differences in female personality. In our study, it might also be the case that birds in some locations are bolder than others and therefore differ in their propensity to produce certain call types (irrespective of predator type). Hence, future work is necessary to investigate if and how mobbing calls in great tits might be affected by personality traits. However, the difference in calling behavior could also be explained by a difference in predation risk caused by tawny owls between study sites. Dutour, *et al*.^33^ showed that the calling behavior of great tits increases with the prevalence in a predator’s diet. Even though we know that tawny owls are present in all our study locations, great tits in some locations might still be more prone to predation by this predator type and hence differ in their calling behavior. Therefore, future studies might analyze the diet composition of predators at specific study locations and relate them to the calling behavior of the prey species in those areas rather than estimating predation risk simply by the presence or absence of the predator species.

We show that great tits vocally discriminate between two common predators, sparrowhawk and tawny owl, that greatly differ in threat level. We further found that great tits use the interval between elements in addition to already known ways^23^ to encode information about predator threat. Furthermore, the number of conspecifics affected the number of uttered calls, which indicates that some community features, such as e.g. familiarity among flock members or flock size, might affect the mobbing behavior of great tits.

## Methods

### Study location

We studied great tits (*Parus major*) near Tübingen (48°31′N, 9°3′E), Freudenstadt (48°27′N, 8°25′ E), and Rottenburg am Neckar (48°28′N, 8°56′E), Baden-Württemberg in southwest Germany. Because a minimum distance of 200 to 250 meters is often used to ensure independent measures in free-ranging parids^14,53^, we usually kept a minimum distance of at least 220 m between study sites (mean ± SE, 616.4 m ± 81.5). In some of our study locations (n = 7), however, the population density of great tits is quite high (25–30 breeding pairs per square-kilometer) (personal observations). Hence, in those areas we could decrease the minimum distance to 170 m (192.7 m ± 7.8) between predator presentations while still keeping the probability of testing the same individual twice low. During all sound recordings, there was a minimum distance of 8 meters between the observer and the microphone.

### Mobbing call recordings

Recordings were made by NK & FA and took place between 07:00 and 14:00 CET from late June 2017 to early April 2018. We used different taxidermy mounts of two different tawny owls (*Strix aluco*; *N* = *2*) and sparrowhawks (*Accipiter nisus, N* = *2*) to elicit mobbing calls. Mounts were placed on tree trunks or rocks and we used only one mount per site. We recorded calls using a boundary microphone (Marantz professional, in Music GmBH, Ratingen, Germany,) placed directly beside the predator model and a digital recorder (Marantz professional PMD661MKIII, inMusic GmbH, Ratingen, Germany). The observer noted the location, model number and time at the start of each recording. Recordings started immediately after setting up the equipment and were terminated 10 minutes after a great tit started to utter mobbing calls. In cases where no great tit participated in mobbing recordings were terminated after 30 minutes. The observer noted the number of conspecifics in a radius of 50 m around the taxidermy mount. In total we recorded mobbing calls at 49 different locations (tawny owl n = 23, sparrow hawk n = 26). However, some of the recordings had poor quality (n = 2), great tit calls strongly overlapped with other bird calls (n = 5), observations got interrupted by pedestrians (n = 2) and in one case a free-living sparrowhawk flew by. Hence those recordings could not be analyzed resulting in a final sample size of 40 (tawny owl n = 19, sparrow hawk n = 21).

### Call analysis

Sound recordings were analysed by NK in a strictly blinded fashion. One of our colleagues (AR) copied all sound files and renamed them with numbers, so there was no reference to location or treatment (tawny owl vs. sparrowhawk). Files were analyzed using Avisoft SASLabPro with a sample rate of 44.1 kHz. We created a sonogram using the Hann window function (FFT length 1024, Frame size 25% and 98,43% overlap). First, we analysed all calls produced by great tits within three minutes of the onset of mobbing. We manually selected calls and visually categorized them into one of four call types (D, jar, chirp or tonal) following the description given by Carlson, *et al*.^23^. Afterwards we analyzed the first five calls of each recording and measured four acoustic parameters: the duration (s) of the call, the duration (s) of each element, the number of elements per call and the interval (s) between elements.

Furthermore, we used a power spectrum analysis (FFT = 512) to determine more detailed analyses of the acoustic features of D elements as described in Templeton, *et al*.^16^. Analyses were performed in the center of the first D element of each of the first five calls of a recording. We only used recordings of very high quality, i.e. the first five calls did not overlap with calls of other birds or any other background noise (tawny owl: n = 16, sparrowhawk: n = 16). In two mobbing events with the tawny owl mount, great tits produced only two mobbing calls, which were also included in the analysis. We measured six spectral features (for details see Templeton, *et al*.^16^): the peak frequency (P), the maximum frequency (M), the number of peaks above −10 dB, the highest (U) and lowest frequency (L) peak above –10 dB relative to the peak, the first (F1) and second (F2) peak above −30 dB. Further, we determined the bandwidth at −10 dB and −30dB by subtracting L from U and F1 from M respectively. The interval between overtones was calculated by subtracting F1 from F2.

### Ethical note

This study was performed in accordance with relevant guidelines and regulations for nature conservancy in Germany (§44 Abs. 1 Nr. 2 BNatSchG). Field observations and mobbing experiments were in accordance with the higher nature conservation authority in Tübingen and adhered to the Guidelines for the Use of Animals in Research of the Animal Behavior Society/Association for the Study of Animal Behaviour.

### Statistical Analysis

We used SAS JMP 16 for data analysis. Before conducting any further analysis, we calculated the mean value of calls per location for all measured response variables.

First, we used t-tests to do a pairwise comparison of the vocal responses (i.e. number of elements, call duration, element duration and interval between elements) towards the two different mount exemplars per predator species (N = 2 tawny owl, N = 2 sparrowhawk) used during this study. By doing so, we tested for differences in vocal responses within treatment groups (i.e. tawny owl and sparrowhawk) that might be provoked by differences in mounts (as they slightly differed in size and color). In cases where data did not show equal variances, we used welch-tests. None of our measured variables differed significantly between the respective two taxidermy mounts (all p > 0.05), i.e. vocal responses did not differ according to which sparrowhawk mount or tawny owl mount was used. Hence, we pooled the data for further analysis into two categories: sparrowhawk versus tawny owl. Moreover, none of our measured variables was significantly affected by observer (all p > 0.05), i.e. vocal responses did not differ according to who of the two observers recorded the audio file. Hence, we did not include observer as a factor in further analysis.

Secondly, we used ANOVAs to test if the number of calls and call types produced within three minutes are affected by the fixed factors predator type, season (i.e. winter (December–February), spring (March–May), summer (June–August) and autumn (September–November)) or number of conspecifics in a radius of 50 meters (henceforth number of conspecifics). We also added location as random variable to the model. Additionally, we calculated call rate (D calls/individual/minute) and conducted and ANOVA including the above-mentioned factors.

Further, we analyzed if the structure of D calls differed between predator types using ANOVAs. We defined number of elements, call duration, element duration and interval between elements as response variables and included predator type, season and number of conspecifics as fixed factors. Lastly, we added location as a random factor to the model.

To test for differences in acoustic features, i.e. peak frequency, maximum frequency above −30 dB, number of peak above −10 dB, interval between overtones, bandwidth at −10 dB and bandwidth at −30 dB, in response to the two predator types we performed ANOVAs including treatment (sparrowhawk vs. tawny owl) and number of conspecifics as a fixed and location as a random factor.

## Data Availability

The datasets generated and/or analyzed during the current study are available from the corresponding author on reasonable request.
